# Sensorimotor strategies for recognizing geometrical shapes: a comparative study with different sensory substitution devices

**DOI:** 10.3389/fpsyg.2015.00679

**Published:** 2015-06-09

**Authors:** Fernando Bermejo, Ezequiel A. Di Paolo, Mercedes X. Hüg, Claudia Arias

**Affiliations:** ^1^Centro de Investigación y Transferencia en Acústica (CINTRA), Universidad Tecnológica Nacional - Facultad Regional Córdoba, Unidad Asociada de CONICETCórdoba, Argentina; ^2^Facultad de Psicología, Universidad Nacional de CórdobaCórdoba, Argentina; ^3^Ikerbasque, Basque Foundation for ScienceBilbao, Spain; ^4^Department of Logic and Philosophy of Science, IAS-Research Center for Life, Mind, and Society, University of the Basque CountrySan Sebastián, Spain; ^5^Department of Informatics, Centre for Computational Neuroscience and Robotics, University of SussexBrighton, UK; ^6^Consejo Nacional de Investigaciones Científicas y Técnicas (CONICET)Argentina

**Keywords:** sensorimotor contingencies, sensorimotor approach to perception, sensory substitution, human echolocation

## Abstract

The sensorimotor approach proposes that perception is constituted by the mastery of lawful sensorimotor regularities or sensorimotor contingencies (SMCs), which depend on specific bodily characteristics and on actions possibilities that the environment enables and constrains. Sensory substitution devices (SSDs) provide the user information about the world typically corresponding to one sensory modality through the stimulation of another modality. We investigate how perception emerges in novice adult participants equipped with vision-to-auditory SSDs while solving a simple geometrical shape recognition task. In particular, we examine the distinction between apparatus-related SMCs (those originating mostly in properties of the perceptual system) and object-related SMCs (those mostly connected with the perceptual task). We study the sensorimotor strategies employed by participants in three experiments with three different SSDs: a minimalist head-mounted SSD, a traditional, also head-mounted SSD (the vOICe) and an enhanced, hand-held echolocation device. Motor activity and fist-person data are registered and analyzed. Results show that participants are able to quickly learn the necessary skills to distinguish geometric shapes. Comparing the sensorimotor strategies utilized with each SSD we identify differential features of the sensorimotor patterns attributable mostly to the device, which account for the emergence of apparatus-based SMCs. These relate to differences in sweeping strategies between SSDs. We identify, also, components related to the emergence of object-related SMCs. These relate mostly to exploratory movements around the border of a shape. The study provides empirical support for SMC theory and discusses considerations about the nature of perception in sensory substitution.

## Introduction

Sensorimotor and enactive theories propose that perception is intimately related to action. In a strong sense, perception is actively constituted by the cognitive agent who engages in perceptually guided action (Varela et al., 1991), which involves both actually executed acts as well as existing expertise and sensibility to action possibilities afforded by the world. In particular, for sensorimotor approaches, perception is based on the regularities that govern the ongoing coupling between action and subsequent sensory changes, also known as sensorimotor contingencies (SMCs). SMCs depend on specific bodily characteristics and kinds of action possibilities that the environment enables and constrains (O'Regan and Noë, 2001; Noë, 2004).

In accordance with O'Regan and Noë (2001) what accounts for the differences between perceptual modalities is the agent's mastery of the structure of SMCs. These regularities can broadly be classified into two types: *apparatus-related SMCs*, which relate to the dynamical and morphological properties of the sensorimotor apparatus that enables certain types of movements and sensory information, and *object-related SMCs*, which arise from the structure of the objects of perception and are associated with the categorization of objects and events in the environment. Performing complex tasks, such as recognizing an object, always involves both types of SMCs in a complex dynamic relationship. This relationship can be hard to disentangle and so far has not been studied in detail.

One way to examine the relation between these different kinds of SMCs is through the use of sensory substitution devices (SSD). Sensory substitution refers to the phenomenon by which environmental information typically acquired through one sensory modality can be obtained by another. For example, visual information can be provided by auditory or tactile stimuli. The term “substitution” must not always be taken literally (as we discuss later) and in the current context it refers to a device that allows a task to be carried out successfully through an atypical sensory modality. According to Lenay et al. (2003), SSDs make it possible to follow with precision the constitution of a new kind of perceptual modality in adults equipped with these devices for the first time. Research into sensory substitution has provided significant empirical support for sensorimotor theories of perception (Hurley and Noë, 2003). These interfaces define new potential actions and sensory inputs and so perception can only be possible by actively establishing appropriate sensorimotor skills: “only through self-movement can one test and so learn the relevant patterns of sensorimotor dependence” (Noë, 2004, p. 13).

According to Myin and Degenaar (2014), evidence obtained in sensory substitution is in agreement with sensorimotor theories of perception. It has been found that after practice with SSDs users skillfully employ the new sensorimotor configuration to resolve perceptual tasks that normally involve the unavailable modality. In some cases the resulting sensorimotor strategies resemble those of the replaced modality. For instance, in the case of vision, those who are allowed to use the SSD to actively explore the world sometimes claim to get a sense that the system is providing visual-style access to the world, thus permitting guided locomotion and object localization using strategies such as estimating distances through parallax cues (Guarniero, 1974, 1977). However, depending on the SSD and configuration, success in similar tasks, while demanding an active control by the subject in order to master the novel sensorimotor situation as predicted by the theory, do not always result in strategies and experiences that directly correspond to the “substituted” modality in a straightforward manner (see e.g., Deroy and Auvray, 2014).

The purpose of the present study is to explore how novel sensorimotor mastery (which according to sensorimotor theory is constitutive of perception) emerges in novice adult participants equipped with different vision-to-auditory devices while solving the task of recognizing simple geometrical shapes. We study the learned sensorimotor strategies by measuring performance, recording trajectories and first-person data. Comparisons between the performance and perceptual trajectories with each SSD allow us to identify what features of the sensorimotor strategies can be associated primarily with the different devices, and which ones can be associated with the task. We expect that differences in the learned sensorimotor strategies used with each device can be attributed to the emergence of apparatus-based SMCs as the use of the SSD is mastered. And, because we maintain a functional equivalence in the shape recognition task, the use of similar strategies would suggest the presence of object-related SMCs. We conjecture that the differences in the sensorimotor strategies between the different devices correspond to the development of new sensorimotor patterns related to the specific requirements of each device while similarities in sensorimotor strategies correspond to preexisting sensorimotor mastery related to the substituted modality.

### Background

There is considerable empirical support for enactive and sensorimotor theories of perception (see, e.g., McGann et al., 2013; Bishop and Martin, 2014). However, the systematic relation between action, perception and sensorimotor dynamics is still poorly understood. Phrases like “mastery of the laws of SMCs” have been given various, sometimes conflicting, interpretations. The original authors, for example, suggest that mastery consists in the acquisition of knowledge at the personal level (accessible to action planning) about the nature of subpersonal processes (SMCs), for instance the knowledge that the projection of a stationary object on the retina would move one way when moving one's eyes to the left, and the other way when moving to the right (O'Regan and Noë, 2001, p. 949). Now, some argue that the notion of knowledge in SMC theory should be abandoned, in favor of the idea that the enactment of SMCs themselves suffices to account for the qualitative differences (between objects, modalities) in perceptual experiences (Hutto, 2005). Others emphasize that perceptual experience in the absence of overt movement can only be explained by reference to the deployment of acquired knowledge (Roberts, 2009).

Our focus in this paper, however, is not to resolve this issue but to study a more practical unresolved question about the kinds of SMCs involved in different perceptual tasks. To the best of our knowledge, Maye and Engel (2012) carried out one of the few attempts to study these different kinds of SMCs empirically using a robotic platform. They proceeded from the assumption that the different types of SMCs are associated with different time scales. The *apparatus-related SMCs* capture the instantaneous effects of actions on the patterns of sensory stimulation, while the *object-related SMCs* account for sequences of actions and sensory observations, that is, some form of exploration. They implemented this distinction in a computational model of SMCs that demonstrated the emergence of perceptual capabilities in a mobile robot. The authors showed how both SMC types determine the overt behavior of the agent: *apparatus-related SMCs* let the agent move in a coordinated and energy-efficient manner and *object-related SMCs* support its behavior adapted to the specific environmental requirements.

Sensory substitution research could potentially help generate more systematic knowledge about the role played by the different kinds of SMCs. To this date, however, a good part of the work in this area has focused on the question of what perceptual modality is present in the experience of a skillful user of a SSD. Some researchers postulate that perception achieved with these devices is similar to the modality being substituted. With a SSD that provides visual information, the SMCs recreated by users would be visual-like, involving relations of distance, relative position between objects and so on, although some visual features (colors) will be absent (O'Regan and Noë, 2001; Hurley and Noë, 2003; Noë, 2004; Myin and O'Regan, 2008). In contrast, others propose that perception remains within the substituting modality. In such a case, the users would simply learn to re-signify auditory or tactile inputs (Humphrey, 1992; Block, 2003; Prinz, 2006). A third position, recently proposed, suggests that sensory substitution gives the user a brand-new mode of perception, not fully reducible to that of any pre-existing sensory modality (Auvray and Myin, 2009; Kiverstein and Farina, 2012; Farina, 2013; Deroy and Auvray, 2014). Despite the discrepancy between these views, no systematic effort has been undertaken to analyze the structures of SMCs involved (O'Regan et al., 2005).

In this regard, it is worth noting the work done with simplified substitution devices or minimalist SSD, whose design offers the minimal technical conditions necessary to enable the perception of certain object properties, e.g., shape and location in space. Because the devices reduce information to a bare minimum, users cannot infer spatial properties on the basis of the sensory stimulation alone. They are forced to deploy a perceptual activity in the form of bodily movements that can easily be observed and recorded, that are called perceptual trajectories (Lenay and Steiner, 2010). Minimalist SSDs illustrate how the constitution and mastery of spatial notions can be grounded on the basis of the sensorimotor laws (Auvray et al., 2007b).

A minimalist SSD, designed by the Suppléance Perceptive team at the Université de Technologie de Compiègne is the TACTOS system (Hanneton et al., 1999). It allows the user to recognize geometrical shapes presented on a screen by moving a cursor and receiving on/off tactile stimulation when the cursor's receptor field encounters at least one black pixel. The participant is blindfolded and moves the cursor using a stylus on a graphic tablet. The tactile stimulation is delivered to the other free hand. Experiments with TACTOS have demonstrated that users with little training (blind persons or blindfolded adults) can learn to recognize simple shapes (Ammar et al., 2005; Rovira et al., 2014). The analysis of perceptual trajectories reveals the emergence of several SMCs and different efficient sensorimotor strategies during the perception of the target shape (Lenay et al., 2003; Sribunruangrit et al., 2004). Stewart and Gapenne (2004) developed a computer algorithm to model perceptual trajectories employed by participants in order to perceive simple shapes. The modeling process has been constrained and informed by the capacity of human subjects both to consciously describe their own strategies, and to apply explicit strategies; thus, the strategies effectively employed by the human subject have been influenced by the modeling process itself. The great advantage of this kind of models is that it provides an explanation, and not just a description, of the active perception of the human subject. Subsequent studies (Amamou and Stewart, 2006, 2007) propose an automatic method for identifying spontaneous sensorimotor strategies developed by participants based on properties of their movements.

Gapenne et al. (2005) compared the performance of participants by varying the type of feedback provided by the TACTOS: tactile (vibration on the finger), auditory (a beep), visual (a flash on the screen of a PC). The results showed that neither the performance nor the development of a successful sensorimotor strategy was significantly affected by the type of feedback. This supports the thesis that there are basic sensorimotor mechanisms that are not defined by the sensory organs or the apparatus, but are strongly influenced by the properties of the perceived object, i.e., object-related SMCs.

Apart from experiments carried out with minimalist devices, not much work has been done to analyze the SMCs involved in perception using more traditional SSD. Much of the research with SSD is concerned with revealing neurophysiological substrates of sensory substitution (for example Renier et al., 2005; Amedi et al., 2007; Hertz and Amedi, 2014); whereas, behavioral studies are focused on the qualities of perception (e.g., Renier et al., 2006; Kim and Zatorre, 2008; Striem-Amit et al., 2012). Others behavioral studies that do not analyze the movements performed by participants highlight the crucial role played by SMCs in the resulting perception. Such is the case of the work of Auvray et al. (2007a) who investigated the performance in dynamic object localization and recognition experiments of blindfolded participants equipped with an SSD called vOICe. This device converts visual images to auditory soundscapes by associating height to pitch and brightness with loudness in a left-to-right scan that last 1 s (details in Meijer, 1992). Participants received intensive training with the device and then they had to explore the perceptual scenes by moving a hand-held camera. The results showed that they were able to generate information in order to localize and recognize the targets. Along similar lines, a recent piece of work investigates how sensorimotor skills can be shared among different sensory modalities (Levy-Tzedek et al., 2012). The authors compare the performance of sighted participants that had to reach a visual target via vision and via visual-to-auditory SSD. During the test, experimenters changed the sensorimotor coupling rules that participant required to solve the task. Results showed that participants can transfer sensorimotor information between both perceptual conditions. The authors conclude that new sensorimotor information can be generalized across sensory modalities and therefore this information cannot be considered as sensory modality specific.

Not all sensory “substitution” experiments aim at replacing an existing perceptual modality for another. One such sensory augmentation device, the feelSpace belt designed at the University of Osnabrück, provides a novel kind of directional information. This belt mediates the information of the magnetic north via continuous vibrotactile stimulation around the waist. Several investigations were conducted to study the perceptual learning that took place in trained participants with the device (Nagel et al., 2005; Kärcher et al., 2012; Kaspar et al., 2014). In general, results indicate that the belt facilitates navigation and allows the usage of new navigation strategies. On this basis, the authors suggest that novel SMCs can be developed with the feelSpace belt, and they link the participants' performance with the both types of SMCs, apparatus-related and object-related working complementarily.

Everyday perceptual performance in some people provides examples of natural sensory substitution systems, e.g., a blind person self-produced sounds (for example, tongue clicks, cane tapping sounds) with the specific purpose of obtaining echoic information in order to detect, localize and recognize unseen silent objects. This kind of human echolocation is a genuine but unexploited ability that has been considered as a natural visuo-auditory substitution system that humans can learn to use (Arias et al., 2011). In a recent study, Milne et al. (2014) evaluate people's ability to use echolocation to recognize simple shapes of objects. The results show that expert echolocators can use this ability to successfully identify shapes, and also that head movements that they make while echolocating are necessary for their correct identification. The authors compare these kinds of movements with eye movements, or saccades, that a sighted person makes when scanning a scene; they suggest that echolocators use sounds for “tracing the contour” of objects. The use of head movements might be a good candidate to explore apparatus-related SMCs in this case. However, more systematic studies would be needed to clarify this issue.

### Overview of experiments

In order to study the emergence and role played by different kinds of SMCs, we analyze and compare the sensorimotor strategies and first-person data involved in a similar shape recognition task using different SSDs. In Experiment 1 participants are equipped with a minimalist SSD. In Experiment 2 they use a traditional visual-to-auditory SSD, the vOICe device. And in Experiment 3 they use echolocation enhanced by a hand-held device called the Sonic Torch.

**Figure 1:**
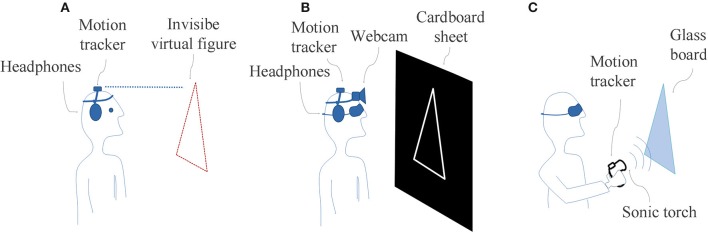
**Illustration of shape recognition task with different auditory-substitution-of-vision devices. (A)** subject equipped with the minimalist SSD (Invisible shapes discoverer.); **(B)** subject equipped with the traditional SSD (vOICe); **(C)** subject assisted with the Sonic Torch (dispositive useful to generate echolocation signals) to solve the task via echolocation skills. Note that in each task the subject has the motion tracker to record his movements.

In Experiment 1, participants must move their head to generate an on-off sound stimulus (Figure 1A); in Experiment 2, they must move their head to generate a complex sound pattern encoded by the device (Figure 1B); and in Experiment 3, they must move a hand to produce echoic information generated by the presence-absence of sound reflections (Figure 1C). In all cases, the movements under study were recorded with a commercial motion tracker (Polhemus Patriot).

All experiments were implemented in perceptually simplified scenarios to reduce the complexity of information available and to improve experimental control. Participants had minimal information about each SSD operation, thus promoting their spontaneous interaction with the experimental setup. They received explanations about which part of their body they had to move in order to interact with the scene and that changes in sound patterns could help them recognize objects. All participants gave informed consent and received monetary compensation for their involvement in each experiment.

We performed pilot studies to determine the size and types of shapes to be used in each experiment in order to the different tasks have a similar difficulty, which was crucial to compare equivalent level of perceptual learning between the tests. The instructions to the participants were the same as the definitive tests except that there was no time limit. In each case, we adjusted the size and type of shapes to ensure that exploratory movements were sufficiently comfortable and precise. We selected for each experiment the set of geometrical shapes that were recognized over chance level and in a reasonable time to avoid fatigue.

## Experiment 1: A minimalist SSD

### Methods

#### Participants

Ten adults with normal hearing (6 men and 4 women) aged between 21 and 30 years (M: 26.1 years, SD: 4.3) completed Experiment 1.

#### Apparatus and materials

The experiment was conducted in a semi-anechoic chamber. Participants were equipped with the minimalist SSD called Invisible shapes discoverer (Figure 1A). It is composed of a motion tracker attached to a plastic headband and headphones (Sennheiser HD530), both connected through software running on a desktop PC (Intel Core 2 Duo @3 GHz; 3.2 GB RAM; Microsoft Windows XP Professional). The SSD uses the elevation and azimuth angles of the participant's head to simulate an invisible virtual geometrical shape in front of the user according to spatial coordinates determined by the position of the motion tracker at the beginning of each trial. Whenever the angular coordinates of the motion tracker make contact with the simulated surface of the shape, a buzz-like sound is emitted via the headphones (Bermejo et al., 2009). The software was developed in MatLab, Mathworks and provides a graphical view of perceptual trajectories, i.e., the complete sequence of head movements (expressed in elevation and azimuth angles) performed by participants in each trial.

The simulated shapes were: a square, a horizontal rectangle, an equilateral triangle and a circle. The surface of the virtual shape was equivalent to a shape of 1600 cm^2^, located at 80 cm from the participant, at the height of the participant's face.

#### Procedure

Participants sat down in the chamber and were equipped with the SSD. The experiment was carried out in the dark. On each trial, the software simulated the invisible geometric shape located in the frontal plane (Figure 1A). Participants were aware of the set of possible shapes that they could be presented with. Their task was to freely explore the object by moving the head and to identify its shape verbally. Before starting the test, they underwent a familiarization trial. The experimenter supplied feedback about correct or incorrect answers and, in this last case, informed also the correct response.

The session consisted of 20 trials where each of the four shapes appears 5 times. The order of presentation was semi-randomized with the follows restrictions: (1) a given shape could not be presented consecutively more than twice; (2) a given shape could not be presented two times more than any other shape.

The trial ended when the participant recognized the shape or after a maximum time of 3 min. On average, each participant took approximately 50 min to complete a full session.

After each session a semi-structured interview was conducted to collect information of participants' experience. The focus of the interview was on two questions: How did the participant identify the object?, that is, metacognitive data; and what kind of subjective experience was involved in recognizing (or not) the object?, that is, first-person phenomenal data.

#### Data analysis

General performance was evaluated according to two variables: (1) Hits: counted as 1 point for a correct answer and 0 point for an incorrect answer. (2) Duration: time (in seconds) elapsed from the beginning to the end of the trial. Means, standard deviations and percentages of each performance value were obtained. We evaluated the effect of shape and trial order over the mentioned variables. The shapes were a square, a rectangle, a triangle and a circle. To account for learning effects, each performance comprising 20 trials was split into four quarters according to the order of appearance: the first quarter (1/4) corresponding to trials 1 to 5, the second (2/4) to trials 6 to 10, the third (3/4) to trials 10 to 15 and the fourth (4/4) to trials 16 to 20. We analyze the performance with non-parametric tests (Friedman and Wilcoxon tests) and univariate repeated measures ANOVAs. Significant *F*-values (*p* ≤ 0.05) are analyzed with the Bonferroni *post-hoc* test.

To conduct the analysis of the sensorimotor strategies, first we established two general kinds of categories of movement patterns. This was realized from the visual inspection of perceptual trajectories belonging to hits trials and the consideration of the meta-cognitive data – participants' expressions regarding exploration modes useful to solve the task. The categories were:

- Sweeps: these were primarily patterns composed of a sequence of straight movements similar to eye-saccades. These movements allow the participant to scan the object in two ways, either entering and leaving the shape area by the contour or crossing the entire area of the shape. A sweeping movement could be small or large. We classify them as micro-sweeps when they correspond to an angle less than 15°; and as macro-sweeps when the angle is equal to or greater than 15°.
- Exploration mode: participants used two general exploratory movements: one was focusing on specific portions of the shape; the other, going throughout its entire perimeter or area. We classify the movement pattern as General exploration when the movements made by participants with their heads were performed over more than 60% of the shape perimeter or area; and as Focal exploration when these movements were performed over 60% or less of the shape.

**Figure 2:**
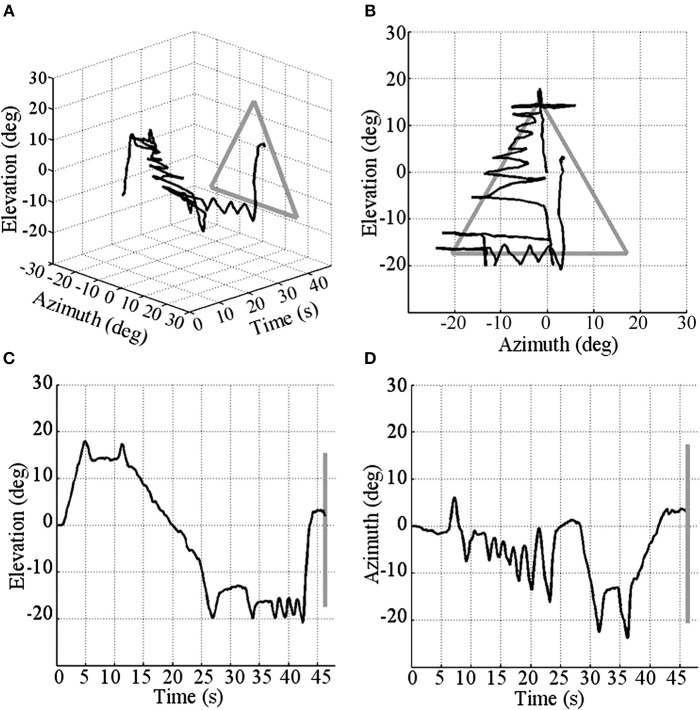
**Perceptual trajectory performed by a participant to recognize a triangle**. In dark gray: perceptual trajectory; in light gray: the geometrical shape. **(A)** 3-D view (elevation, azimuth and time); **(B)** front view (elevation and azimuth); **(C)** side view (elevation and time); and **(D)** top view (azimuth and time).

The combination of these categories allows us to classify different sensorimotor strategies according to the prevalent mode of sweep and exploration used within a given trial as: Macro-General, Macro-Focal, Micro-General, Micro-Focal. When participants used more than one of these sensorimotor strategies in a same trial, we considered it as a Mixed strategy. We analyzed the percentage of use of each of these sensorimotor strategies in successful trials as a function of the geometric shape and the trial order. This process was independently performed by two different coders. Agreement between coders was 93.6% and the inter-rater agreement (kappa) was 0.92 (*p* < 0.000). Figure 2 shows an example of a trajectory corresponding to a Micro-Focal sensorimotor strategy performed by a participant while recognizing a triangle. The participant always uses Micro-sweeps, exploring, at first, the upper region of the shape, i.e., the apex of the triangle; then he partially explores the right border; and finally scans a part of the bottom.

Analysis of phenomenal data was performed from a qualitative analysis of the answers given by the participants during the post-test interview.

### Results and discussion

#### General performance

**Figure 3:**
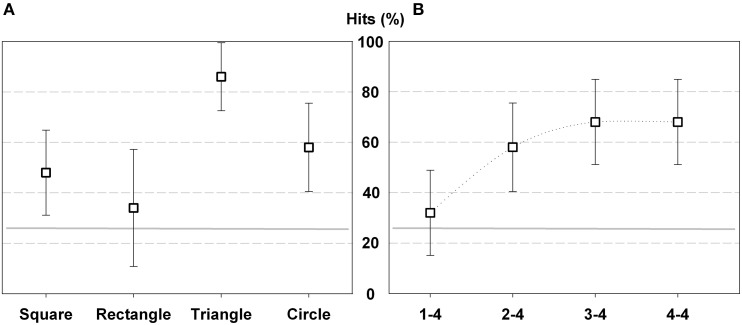
**Percentage of hits in Experiment 1 according geometric shape (A) and trial order (B)**. The gray horizontal line indicates the chance level (25%), and the error bars represent standard deviation (SD).

The analysis of performance showed that hit rates for all participants exceeded chance level. The mean hit rate was 56.5%. The triangle was the most recognized figure (86%), followed by the circle (58%) and the square (48%), while rectangle (34%) was distinguished just above chance level. Hit comparison according to geometric shapes showed significant differences [*F*_(3, 9)_ = 15.017; *p* < 0.000; η^2^ = 0.625]. It was significantly easier to recognize triangle than circle (*p* < 0.05), square (*p* < 0.05) and rectangle (*p* < 0.05) shapes (Figure 3A). The response matrix (Table 1) shows significant differences between hits and confusions for the square (*X*^2^*r* = 14.2, *p* = 0.001), the rectangle (*X*^2^*r* = 12.7, *p* = 0.003), the triangle (*X*^2^*r* = 23.5, *p* < 0.000) and the circle (*X*^2^*r* = 16, *p* < 0.000). In the case of the square, hits exceed confusions square-circle (*Z* = −2.095, *p* = 0.02) and square-triangle (*Z* = −2.871, *p* = 001); but hits do not differ from square-rectangle confusions. For the rectangle, there were more hits than rectangle-triangle confusions (*Z* = −2.54, *p* = 0.01); while hits and rectangle-square and rectangle–circle confusions do not reach significant differences. For the circle, hits exceed circle-square (*Z* = −2.39, *p* = 0.02), circle-rectangle (*Z* = −2.86, *p* = 0.004) and circle-triangle (*Z* = −2.83, *p* = 0.002) confusions. Also in the case of the triangle, the easiest shape, there were more hits than any kind of confusions, triangle-square (*Z* = −2.86, *p* = 0.002), triangle-rectangle (*Z* = −2.84, *p* = 0.002), or triangle-circle (*Z* = −2.83, *p* = 0.002).

**Table 1:** **Response matrix for Experiment 1**.

**Response**	**Shapes**			
	**Square**	**Rectangle**	**Triangle**	**Circle**
Square	*48 (16.87)*	26 (21.19)	6 (13.5)	20 (18.86)
Rectangle	44 (26.33)	*34 (23.19)*	2 (6.32)	20 (21.08)
Triangle	2 (6.32)	6 (9.66)	*86 (13.49)*	6 (9.66)
Circle	16 (18.37)	16 (12.64)	10 (14.14)	*58 (17.51)*

*Mean percentage and SD of hits (italics) and confusions*.

Hit comparison according to trial order also showed significant differences [*F*_(3, 9)_ = 10.965; *p* < 0.000; η^2^ = 0.549]. Participant improved performance as the test progressed, they performed fewer hits in the 1/4 of trials than in the 2/4 (*p* < 0.00), 3/4 (*p* < 0.05) and 4/4 (*p* < 0.00) (Figure 3B).

The mean duration of the trials was 60.06 s (SD 22.8). There were no significant differences in the time that participants took to recognize the different geometric shapes. However, they required fewer mean times to recognize the triangle (50.9 s, SD 23.5) than the others shapes, square (69.62 s, SD 15.38), rectangle (58.24 s, SD 26.4) and circle (61.6 s, SD 17.9). Moreover, participants required on average a similar time to complete the task in the different quarters of trials. We note that in all experimental conditions there was a high inter-subject variability of trial duration.

Several studies demonstrated that shape recognition is possible using the TACTOS, a hand-held SSD similar to the one described above, and that this ability improves with practice (Hanneton et al., 1999; Ammar et al., 2002; Sribunruangrit et al., 2004). Rovira et al. (2010) evaluated the ability to recognize shapes that were similar to those used here but they evaluated blind participants. Similarly to what we saw in our experiment, their results showed that the triangular shapes were the easiest to recognize, followed by curved shapes and quadrilateral and open shapes. Probably, the presentation of two kinds of quadrilaterals shapes (square and rectangle) in our experiment affected the participant performance causing frequent confusion between the two.

#### Sensorimotor strategies analysis

The perceptual trajectories of 109 trials that corresponded to hit responses of all participants were analyzed. As mentioned, the combination of sweeps types and exploration mode offer us 4 kinds of sensorimotor strategies: Macro-General, Macro-Focal, Micro-General and Micro-Focal (see a detailed description of these strategies in Table 2, and examples of each one in Supplementary Material). In some trials, we also identified the use of a Mixed strategy. In these cases, participants recognized the shape with information from one strategy and then confirmed or complemented the reconnaissance with information from another strategy. For example, one participant described the following “At first I tried to get a rough idea about which [shape] it could be, I tried to see how large it was. When I thought I knew, I looked for specific parts of the shape. I looked for a top tip in case it was a triangle, if I didn't find the tip, then it was the circle (Participant 5).”

**Table 2:** **Components of sensorimotor strategies in Experiment 1**.

**Sweeps type**	**Macro**		**Micro**	
**Exploration mode**	**Generalized**	**Focalized**	**Generalized**	**Focalized**
**Sensorimotor Strategies**	**Macro-General**	**Macro-Focal**	**Micro-General**	**Micro-Focal**
Schematic representation	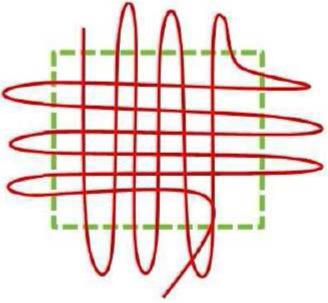	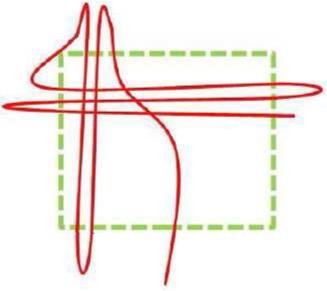	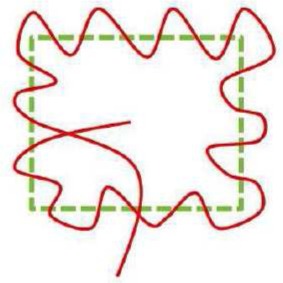	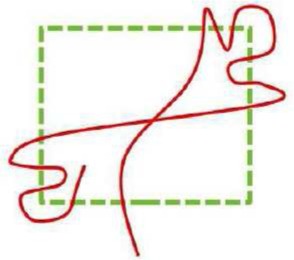
Description	Large head movements crossing the object side to side, exploring its entire area.	Large head movements crossing the object side to side, exploring only parts of its area.	Small head movements going in and out of the object's surface, exploring its entire perimeter.	Small head movements going in and out of the object's surface, exploring only parts of its perimeter.
Metacognitive data	I felt as if I was painting the shape with my gaze. I was filling in the shape as I heard the sounds. So I moved over the shape to paint it whole (Participant N° 6).	It was like painting strokes to uncover the shape. You couldn't make fine moves. I painted a few in some places to find out which shape it was (Participant N° 4).	I moved from the center toward the edges and then I begun to realize what shape it was. I went back and forth many times on all sides (Participant N° 1).	My strategy was to search for the tips of the shape. I started from the outside and moved into the shape. (Participant N° 8).

*Schematic representation (frontal plane) of the perceptual trajectories. Red line: perceptual trajectory; dotted green line: limits of experimental object (Shape: Square)*.

**Figure 4:**
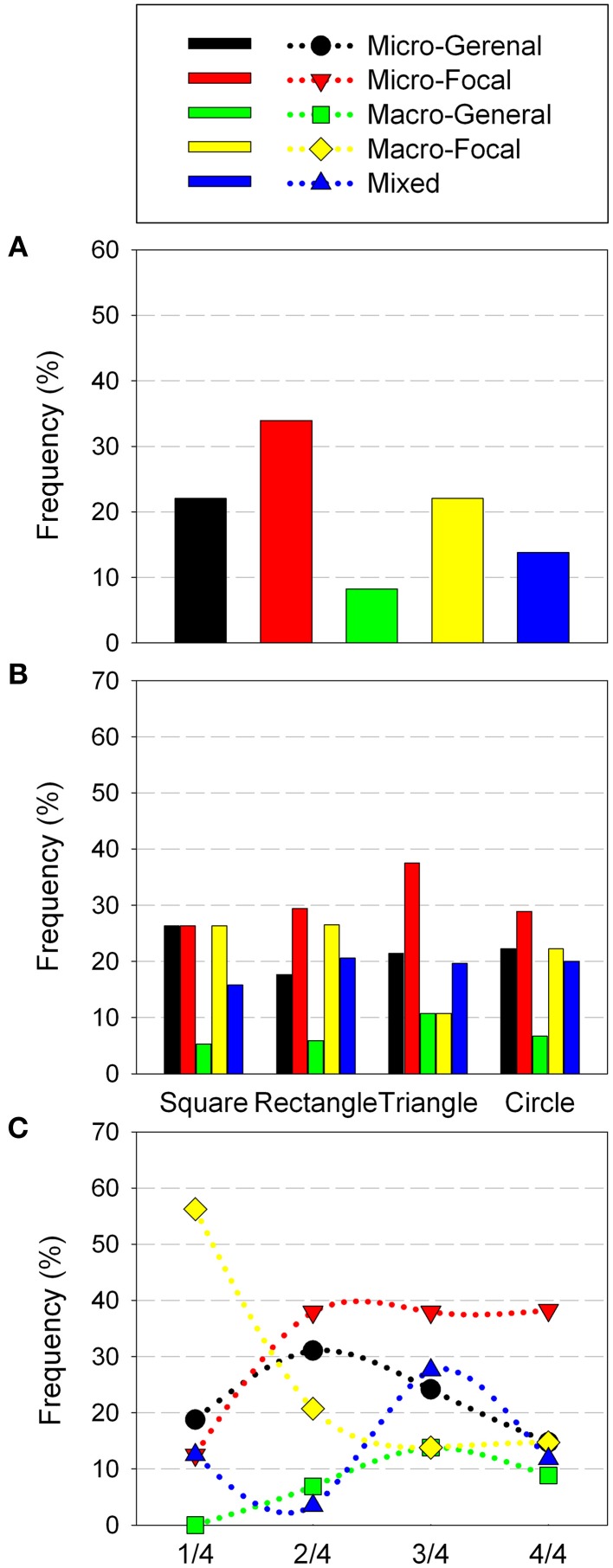
**Sensorimotor Strategies distribution in Experiment 1 according: general use (A) geometric shape (B) and trial order (C)**. The figures report the cumulative percent of strategies of all participants.

For the trials analyzed, most of its perceptual trajectories corresponded to the Micro-Focal strategies (33.9%), then followed the by Macro-Focal and Micro-General strategies (22% in each case). Finally, the remaining trajectories belonged to Mixed (13.7%) and Macro-General (8.2%) strategies (Figure 4A). Differences in the use of sensorimotor strategies did not reach statistically significant level.

Regarding the geometric shapes, the square was mainly recognized using Macro-Focal, Micro-General and Micro-Focal strategies (26.3% for all three). The rectangle was recognized predominantly with focalized exploration strategies either with micro sweeps (29.4%) or with macro sweeps (26.4%). Circle, was specially recognized with Micro-Focal (28.8%), but also with Macro-Focal (22.2%) and Micro-General (22.2%) strategies. Triangle was preferentially recognized with Micro-Focal strategies (37.5%) (Figure 4B). While overall Micro-Focal strategy was the most used, in the square and rectangle Macro-Focal strategy were also widely used. This is probably due to exploration of these quadrilateral shapes through Micro-Focal strategy provides similar information in both cases (presence of straight sides and right angles), which could lead the participant to confuses each other (as shown in Table 1). Sensorimotor strategies with macro sweeps could be useful to solve confusion between the square and the rectangle as they may provide more direct information about the height and width of each shape.

Analysis of sensorimotor strategies according to trial order showed that in 1/4 of trials Macro-Focal strategies were used most frequently (56.2%), and its employment descended in the following quarters. In 2/4, 3/3, and 4/4 of trials strategy most used was the Micro-Focal (37.9, 32.3, and 43.3%, respectively). This sensorimotor strategy was used by participants differently throughout the test (*X*^2^*r* = 10.5, *p* = 0.009), was most employed in the end of the test that in the beginning (*Z* = −2.428, *p* = 0.016). The remaining strategies showed no significant changes along the trials (Figure 4C).

In a comparable shape recognition study with the TACTOS device (Ziat et al., 2007), the following sensorimotor strategies were identified: micro-sweeping (the participant voluntarily oscillates along the shape's contour); macro-sweeping (the participant crosses the line right through); continuous follow-up (the participant tries to maintain a constant contact with the shape); lateral tap (the participant rebounds on the contour without crossing it). Our results show that, on the one hand, the sensorimotor strategies have similar zigzagging movement patterns as are described for micro-sweeping and macro-sweeping strategies. On the other hand, continuous follow-up and lateral tap were not observed here. The reason for these differences is probably the way each device works. The TACTOS emits stimulation only when the user is in contact with the shape contour, not when he/she is in contact with its area, as is the case in Experiment 1. Another difference is the different movement precision allowed by head angular movements vs. a hand-controlled stylus in the case of TACTOS.

#### Phenomenal data

All participants reported that their experience was closely linked to the exploration performed. They felt that the object was being constructed with the sound that appeared and disappeared as they explored the environment. Some participants found it difficult to describe their experiences. However, several participants reported that the progressive way of constructing the object's shape allowed them to imagine themselves “seeing” the object (in Supplementary Material we include typical samples from interviews describing phenomenal experience during shape recognition).

As noted by the participants, shape perception was experienced as emerging as they interacted with the experimental object. It has been described that this kind of devices circumscribes the user to a new perceptual space that emerges at the time they are used (Auvray et al., 2007b; Lenay and Steiner, 2010). The use of visual imagery (painting, pointing with a laser) is probably a resource used by participants to structure the novel kind of information detection.

## Experiment 2: The vOICe device

### Methods

#### Participants

Fourteen adults with normal hearing and vision (7 women and 7 men) aged between 18 and 38 years (M: 24.2 years, SD: 5.7) completed the experiment 2. Two of these participants were excluded from the sample because were not able to establish the minimal necessary skills to solve the task. One participant of Experiment 1 also participated in Experimenter 2, in different days.

#### Apparatus and materials

The experiment was conducted in the same semi-anechoic chamber as in Experiment 1. Participants were equipped with the traditional SSD vOICe. It consists of a portable webcam (Phillips Shs390) and headphones (Phillips SPC 9100/NC14). A desktop PC ran the vOICe software (Learning Edition v1.91) in its default configuration. The vOICe scans the webcam snapshots once per second and from left to right. The device generates a complex stereo sound pattern that lasts 1 s: variations in optical image height are translated as pitch modifications and changes in optical image brightness are encoded as loudness variations (details in Meijer, 1992). The webcam and the motion tracker were attached to a plastic headband.

The software used to administer the test was developed in C++ and C # (Sharp) and its database was implemented on SQL Server 2005 Express. It provides a graphical view of perceptual trajectories.

The experimental objects were a square, a triangle and a rhombus. The surface of each shape was 100 cm^2^. They were made of sheets of cardboard and were supported by a mast, located at 80 cm in front of the participant, at the level of the face. In order to generate a high contrast between figure and background in images transmitted by the webcam, the borders of the shapes were white, while the rest of the figure and the background were black.

#### Procedure

The procedure was similar the one use in Experiment 1. Participants sat down in the chamber, were blindfolded and equipped with the SSD vOICe (Figure 1B). In each trial the experimenter positioned the experimental object in front of the participant. The participants' task was to freely explore the object moving the head and to identify its shape verbally. Initially, they underwent a brief practice of 30 min, to develop minimal abilities to handle the vOICe (described in Supplementary Material). As in experiment 1, the experimenter gave feedback about correct or incorrect responses.

The session consisted of 18 trials where each of the three shapes appears 6 times. The order of presentation was semi-randomized as Experiment 1. The trial ended when the participant recognized the shape or after a maximum time of 3 min. On average, each participant took approximately 90 min to complete a full session. To avoid fatigue, two pauses were held, after the practice and at the middle of the test. Like in Experiment 1, a semi-structured interview was conducted to collect information of participants' experience.

#### Data analysis

General performance, sensorimotor strategies and phenomenal data were analyzed as in Experiment 1. In this case, geometric shapes were a square, an equilateral triangle and a rhombus. In the evaluation of sensorimotor strategies, the agreement between coders was 93.5% and the inter-rater agreement (kappa) was 0.89 (*p* < 0.000). Each performance comprising 18 trials was split into 3 thirds according to the order of appearance: the first third (1/3) corresponding to trials 1 to 6, the second (2/3) to trials 7 to 12 and the third (3/3) to trials 13 to 18.

### Results and discussion

#### General performance

**Figure 5:**
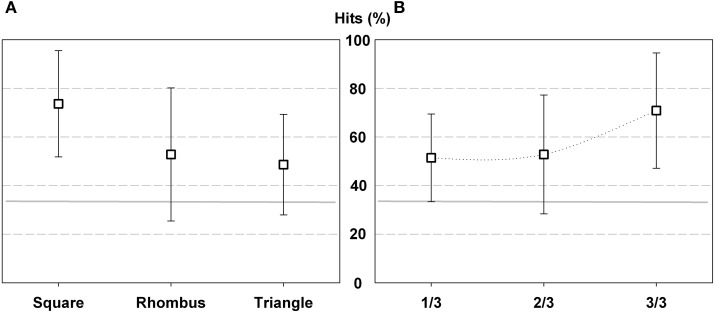
**Percentage of hits in Experiment 2 according geometric shape (A) and trial order (B)**. The gray horizontal line indicates the chance level (33%), and the error bars represent SD.

The analysis of performance showed that hit rates of all participants exceeded chance level. The mean hit rate was 58.8%. The square was the most recognized figure (73.6%), while the hit rate of the diamond and triangle was lower (52.8 and 48.6%, respectively). Hit comparison according to geometric shapes showed significant differences [*F*_(2, 24)_ = 4.918; *p* < 0.017; η^2^ = 0.309]. For the participants was significantly easier to recognize square than rhombus (*p* < 0.05) and triangle (*p* < 0.05) shapes (Figure 5A). The response matrix (Table 3) shows significant differences between hits and confusions for the square (*X*^2^*r* = 16.7, *p* < 0.000), rhombus (*X*^2^*r* = 6.9, *p* = 0.03), and the triangle (*X*^2^*r* = 10.6, *p* = 0.003). In the case of the square, the easiest shape, hits exceed square-rhombus confusions (*Z* = −2.95, *p* = 0.001) and square-triangle confusions (*Z* = −2.87, *p* = 0.002). For the rhombus, there were more hits than rhombus-square confusions (*Z* = −2.73, *p* = 0.005); while hits and rhombus-triangle confusions do not reach significant differences. For the triangle, hits exceed triangle-rhombus confusions (*Z* = −2.49, *p* = 0.009), but not the triangle-square confusions. The greater ease to recognize the square is possibly related to the distinctive soundscape that its shape generated when was scanned by the vOICe. While the oblique lines of the rhombus and the triangle provoke upstream and downstream pitch modulations; the square soundscape, as it contains only vertical and horizontal lines, did not have that kind of modulation.

**Table 3:** **Response matrix for Experiment 2**.

**Responses**	**Shapes**		
	**Square**	**Rhombus**	**Triangle**
Square	*73.61 (21.5)*	11.11 (16.41)	15.27 (20.66)
Rhombus	13.88 (17.16)	*52.77 (27.37)*	33.33 (29.3)
Triangle	37.5 (18.96)	13.88 (19.89)	*48.61 (20.66)*

*Mean percentage and SD of hits (italics) and confusions*.

Hit comparison according trials order also showed significant differences [*F*_(2, 24)_ = 3.903; *p* < 0.035; η^2^ = 0.262]. Participants performed fewer hits in the 1/3 and 2/3 of trials than in the last third of trials (*p* < 0.05), which suggest that they improved performance at the end of the test (Figure 5B).

The mean duration of the trials was 82.38 s (SD 17.97). Trial duration was not affected by the type of geometric shape. As testing progressed participants required less time to solve the task. However, this trend was not statistically significant.

The results are in the same direction as the study of Auvray et al. (2007a) in which participants using the vOICe were able, after a training of 15 h., to recognize familiar objects and discriminating those who belonged to the same category. Brown et al. (2011) evaluated blindfolded sighted participants in a geometrical shape recognition task. They assessed if participant could identify the shape (equilateral triangle, square, rhombus and circle) through just listening its pre-recorded soundscapes images obtained with the vOICe, with no explicit instructions about how the device works. The participant's performance reaches around 33% of hits (with a chance level of 25%) in 72 trials. The authors explained the poor results obtained because of the lack of body movements, indispensable to develop perceptive abilities in this type of tasks. Participants in our experiment reach 57% of hits (with a chance level of 33%) in just 18 trials. The possibility of moving freely could explain the better performance of participants in the present experiment.

Others studies with a visual-to-auditory SSD called PSVA showed that blind and sighted participants were able, after a training period of 10–12 h., to recognize simple figures on a computer screen (Arno et al., 1999, 2001). In these experiments, participants' performance improved significantly with practice. The authors remarked that performing head movement was a fundamental behavior to acquire abilities to recognize that shapes.

#### Sensorimotor strategies analysis

The movement trajectories of 107 trials corresponded to hit responses were analyzed. As in Experiment 1, the combination of sweeps types and exploration mode defined four kinds of sensorimotor strategies: Macro-General, Macro-Focal, Micro-General, and Micro-Focal. However, unlike the trajectories observed in Experiment 1, here it was observed that head movements were mostly like Micro-Sweeps, with low amplitude (<10° approximately) and with fixations well defined (relative motionlessness states) between each one (see a detailed description of these strategies in Table 4, and examples of each one in Supplementary Material). In this experiment, Mixed strategies were not observed.

**Table 4:** **Sensorimotor strategies components of that used Micro-Sweep**.

**Sensorimotor strategies with micro sweeps**	**Micro-General**	**Micro-Focal**
SMCs Object-related	Generalized Exploration	Focalized Exploration
Schematic representation	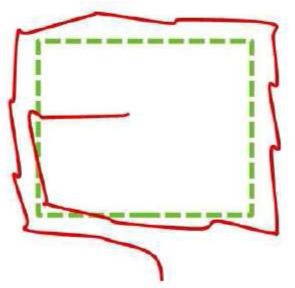	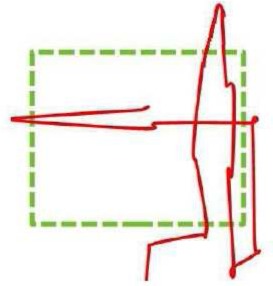
Description	Small head movements through the perimeter of the figure or surrounding it	Small head movements going in and out of the object's surface, exploring only parts of its perimeter
Metacognitive data	*I had told myself to go for the edges* (Participant N° 5)	*I tried to isolate the sounds from different parts because with the whole shape I couldn't tell where the sound was coming from* (Participant N° 13)

*Schematic representation (frontal plane) of the perceptual trajectories. Red line: perceptual trajectory; dotted green line: limits of experimental object (Shape: Square)*.

**Figure 6:**
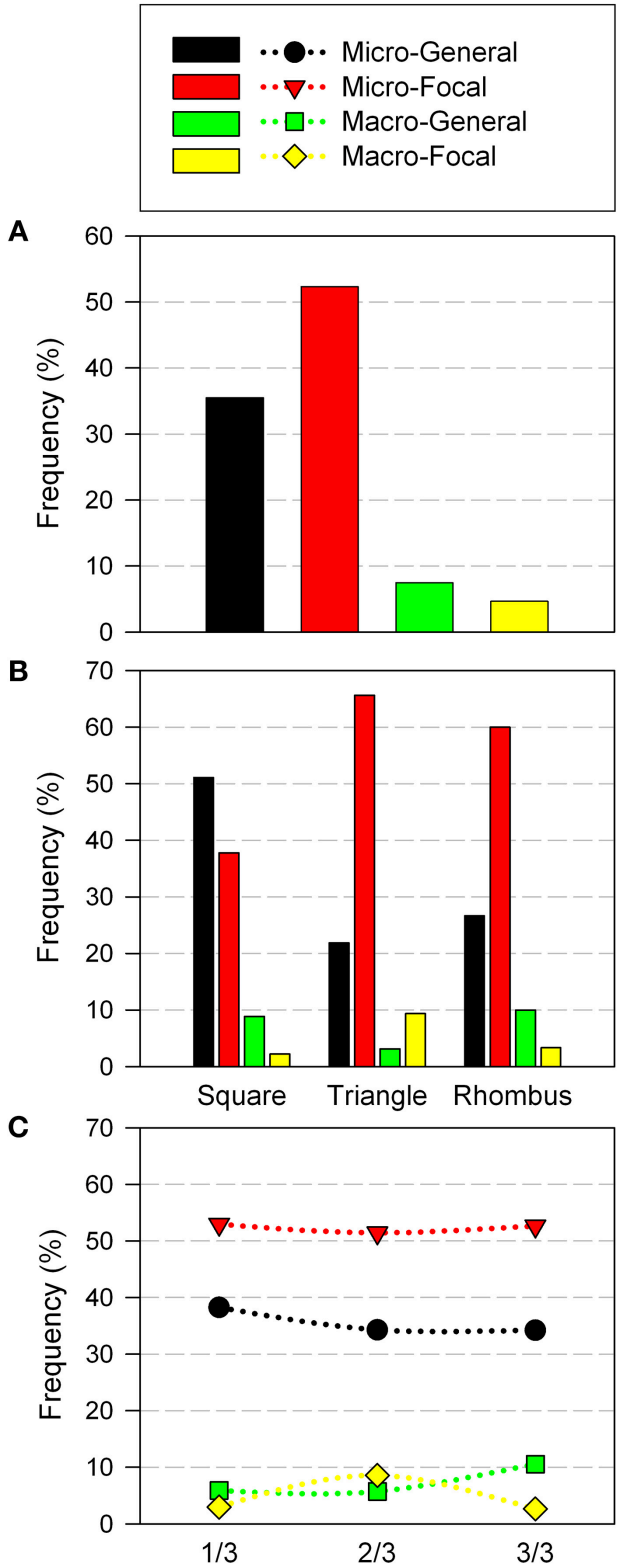
**Sensorimotor Strategies distribution in Experiment 2 according: general use (A) geometric shape (B) and trial order (C)**. The figures report the cumulative percent of strategies of all participants.

Most trajectories correspond to the Micro-Focal strategies (52.34%) and to Micro-General strategies (35.51%). The minority correspond to Macro-General (7.48%) and Macro-Focal (4.67%) (Figure 6A). The use of sensorimotor strategies with micro-sweeps (Focalized and Generalized) was significantly more frequent than the use of strategies whit macro-sweeps (*Z* = −2.594, *p* = 0.003). Only two participants (No. 9 and No. 11) made SM Strategies that included Macro-Sweeps. Like in Experiment 1, the strategy most used was Micro-Focal. Nevertheless, unlike those movement patterns that had a zigzagging trajectory, the directions of these movements were very diverse. In particular, this was observed in the Micro-General strategy, where participants tend to move around the perimeter of the shape, without entering and leaving.

The analysis of sensorimotor strategies according to geometric shapes has shown that the square was mainly recognized using Micro-General strategies (51.1%). Furthermore, the triangle and rhombus were recognized mainly with Micro-Focal strategies (65.6 and 60%, respectively) (Figure 6B). These results are consistent with those found in the general performance. For the square, the easiest shape to recognize, movements around its perimeter highlight their distinctive cues. While recognizing the rhombus and the triangle demands a more detailed examination (i.e., to detect upper and lower angles or an upper angle and a horizontal base).

Analysis of the sensorimotor strategies according to trials order showed that the use of different types of strategies did not change significantly along the test (Figure 6C).

There are not many studies that provide a detailed analysis of the movements that participants make with the vOICe or with other similar SSDs. In their study, Auvray et al. (2007a) remark that participants explored the scene making jerky movements. These kinds of movements, also observed here, are probably related to how the SSD works. Participants learn implicitly that their movements must be coupled to the acquisition and scanning of the optical images. They make fixations that allow the vOICe device to complete the built-in image scan and emit the soundscape; and between each fixation they could make just a short movement. Large and continuous movements would not allow the user to make sense of the sound pattern.

#### Phenomenal data

All participants mentioned that in the task they felt auditory experiences. Some of them recognized the object by global soundscape of each shape. On the contrary, others had to find sound clues throughout the trial (in Supplementary Material we present typical samples from interviews describing phenomenal experience during shape recognition).

Similarly, Auvray et al. (2007a) also found that participants reported principally auditory experiences in recognition tasks with the vOICe. The experiences of those participants, with increasing practice, went from a deductive reasoning to an immediate apprehension, i. e., the vOICe became easier to use and the access to the environment was more direct. Also, some participants were able to quickly recognize some objects by their characteristics sounds. The authors suggested that the participant identified the “sound signature” of each object just as expressions of Global auditory perception observed in this work.

## Experiment 3: Echolocation

### Methods

#### Participants

Seventeen adults with normal hearing and vision (9 women and 8 men) aged between 18 and 38 years (M: 25 years, SD: 6.18) completed Experiment 3. Three of these participants was excluded from the sample because were not able to establish the minimal necessary skills to solve the task. All participants of Experiment 2 also took part in Experimenter 3, in different days. The time order of each experiment was counterbalanced.

#### Apparatus and materials

The experiment was conducted in the same semi-anechoic chamber as in Experiment 1 and 2. Participants were equipped the Sonic Torch (ST), a hand-held tool designed to facilitate sound generation in echolocation tasks (Gilberto et al., 2013). The device emits a semi-directional sound and allows the user to detect nearby objects through listening to the sound changes arisen when the signal is reflected by the object. ST is composed by a high frequency speaker positioned at the end of a plastic tube that emits a constant filtered white noise (band-pass from 400–8000 Hz). The participant has a manual device to switch the system on and off and control the signal intensity. Both ST and the motion tracker were fixed to a wooden handle.

Test administration and the record of hand movements were made with the same software used in Experiment 2. The experimental objects were a square, a triangle and a circle. They were made of glass board and were supported by a mast covered with phono-absorbent material, located 80 cm in front of the participant, at the height of his chest.

#### Procedure

As in Experiments 1 and 2, participants sat down in the chamber, were blindfolded and held the ST with one hand (Figure 1C). On each trial, the experimenter positioned the experimental object in front of the participant. Their task was to freely explore the object moving the hand and to identify its shape verbally. As before, the experimenter provided feedback about corrects responses. Before starting the test, participants underwent a familiarization trial.

The session consisted of 18 trials presented in a semi-randomized order, where each of the 3 shapes appears 6 times. The trial ended when the participant recognized the shape or after a maximum time of 3 min. On average, each participant took approximately 70 min to complete a full session. To avoid fatigue, one pause was held at the middle of the test.

After each session a semi-structured interview was conducted to collect first-person phenomenal data.

#### Data analysis

General performance, sensorimotor strategies and phenomenal data were analyzed as in the previous Experiments. In this case, the shapes were a circle, a square and an equilateral triangle. In the evaluation of sensorimotor strategies, the agreement between coders was 92.4% and the inter-rater agreement (kappa) was 0.90 (*p* < 0.000). Each performance was split into 3 thirds, as in Experiment 2: the first third (1/3) corresponding to trials 1 to 6, the second (2/3) to trials 7 to 12 and the third (3/3) to trials 13 to 18.

### Results and discussion

#### General performance

**Figure 7:**
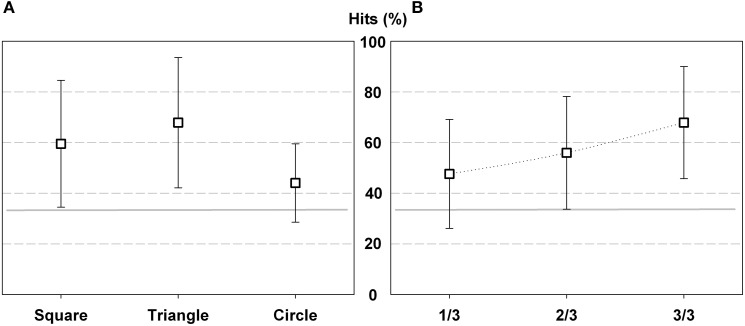
**Percentage of hits in Experiment 3 according geometric shape (A) and trial order (B)**. The gray horizontal line indicates the chance level (33%), and the error bars represent SD.

As in Experiment 1 and 2, the Hit rates for all participants exceeded chance level, mean hits rate was 57.14%. The triangle and square were widely recognized (67.8 and 59.5% respectively), while the circular shape was distinguished in fewer trials (44%). Hit comparison according geometric shapes showed significant differences [*F*_(2, 26)_ = 5.061; *p* < 0.014; η^2^ = 0.28]. As in Experiment 1, it was significantly easier for participants to recognize the triangle than the circle (*p* < 0.00) (Figure 7A). The response matrix (Table 5) shows significant differences between hits and confusions for the square (*X*^2^*r* = 12.7, *p* = 0.001) and the triangle (*X*^2^*r* = 18.6, *p* < 0.000). In the case of the square, hits exceed confusions square-circle (*Z* = −2.35, *p* = 0.02) and square-triangle (*Z* = −2.94, *p* = 001). For the triangle, there were more hits than triangle-square confusions (*Z* = −3.08, *p* < 0.000) and triangle-circle confusions (*Z* = −2.78, *p* = 0.003). In the case of the circle, hits exceed all circle-triangle confusions (*Z* = −2.39, *p* = 0.02), but not the circle-square confusions.

**Table 5:** **Response matrix for Experiment 3**.

**Responses**	**Shapes**		
	**Square**	**Triangle**	**Circle**
Square	*59.52 (25.07)*	11.9 (15.23)	28.57 (17.81)
Triangle	9.52 (14.19)	*67.85 (25.7)*	22.61 (19.17)
Circle	29.76 (17.51)	26.19 (15.62)	*44.04 (15.48)*

*Mean percentage and SD of hits (italics) and confusions*.

Hit comparison according trials order did not show significant differences. However, the percentage of hits increases in relation to each third of trials. From the total hits, 47.6% (SD 21.5) corresponded to 1/3 of trials, 56% (SD 22,2) to 2/3 of trials and finally 67.9% (SD 22.1) to 3/3 of trials. This result indicates that as the test progressed, participants' performance improves (Figure 7B).

The mean duration of the trials was 109.6 s (DS 45.7). Trial duration was not affected by the geometric shape of objects. We note that trials showed a similar mean duration and standard deviation across shapes. Participants required on average a similar time to complete the task in the different thirds of trials.

The results of the scarce literature on the subject of object recognition through human echolocation are in similar direction as the results mentioned. We can mention the work of Rice (1967) and of Hausfeld et al. (1982) who found that blind and sighted blindfolded participants distinguished geometrical shapes (circle, triangle, and square) with fair reliability in close distances (25 cm from the face). Participants in both studies emitted oral signals and moved their heads to trace the edges of the forms presented. The authors suggested that this sort of echo perception requires very little training.

#### Sensorimotor strategies analysis

Perceptual trajectories of 132 trials that corresponded to hit responses were analyzed. Movements patterns performed with the hand were the same as the patterns of head movements described in previous experiments (see metacognitive data corresponding to each strategy in Table 6, and examples in Supplementary Material).

**Table 6:** **Metacognitive data of sensorimotor strategies in Experiment 3**.

**Sensorimotor Strategies**	**Macro-General**	**Macro-Focal**	**Micro-General**	**Micro-Focal**
**Metacognitive data**	*I tried to delimit the frame of the shape using the sound inside the shape and the sound outside … Every time I passed over the shape, across its width, I would be able to tell how much time the hand was passing over it … It was like making brushstrokes to see the duration, I say brushstrokes because the movement was similar* (Participant N° 2)	*[Commenting on the exploration of a triangle]. The sound would cut short faster here [at the top] and slower down here [at the bottom]. If it were a triangle, the sound would cut off at the top. Not below, where the sound was prolonged* (Participant N° 9)	*I tried to find the border; once I found it, I tried to follow it to see whether it would end in a straight line, or whether it would go on, in a straight direction or otherwise* (Participant N° 11)	*At the top and on the right I could find the edges well. When I found an edge, I tried to move really close to it to see what it would do, which way the shape would go* (Participant N° 13)

**Figure 8:**
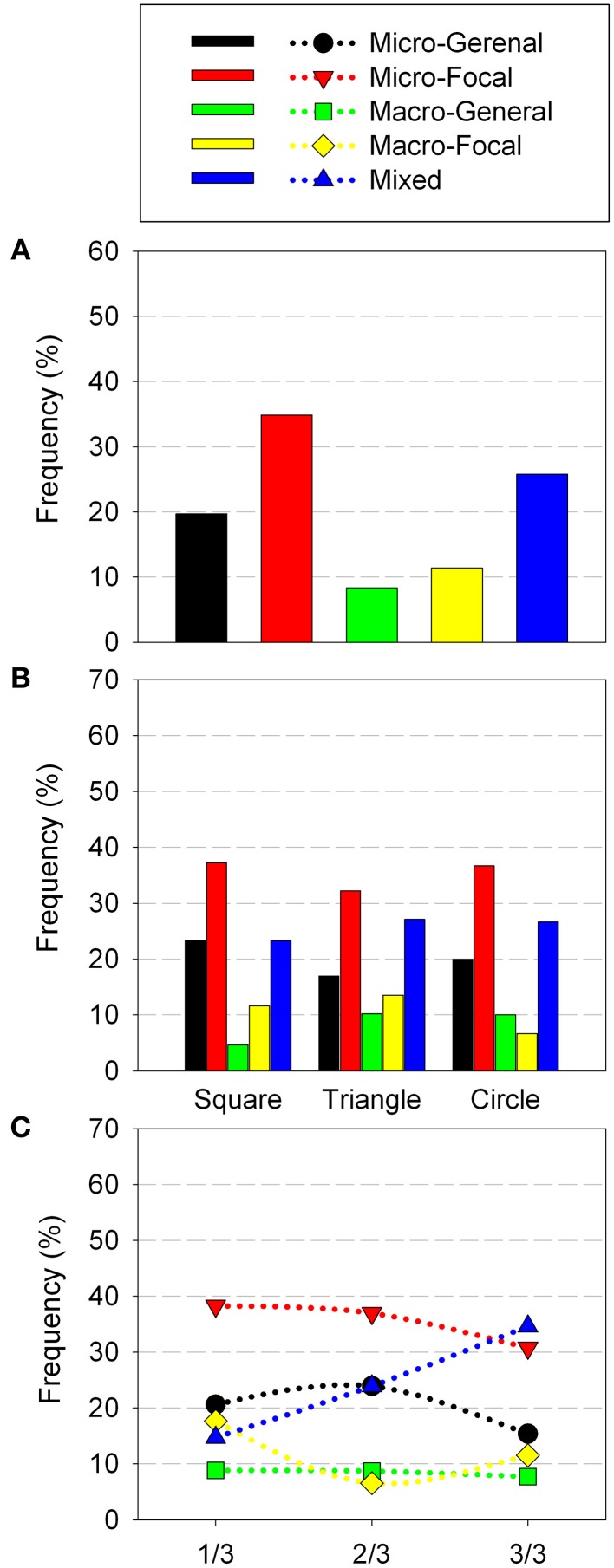
**Sensorimotor Strategies distribution in Experiment 3 according: general use (A) geometric shape (B) and trial order (C)**. The figures report the cumulative percent of strategies of all participants.

Most trajectories correspond to Micro-Focal strategies (34.8%), followed by Mixed strategies (25.7%) and Micro-General strategies (19.7%). Finally, the remaining trajectories belong to Macro-Focal (11.3%) and Macro-General (8.3%) strategies. Participants employed more frequently the Micro-Focal strategy than the Macro-General and Macro-Focal (*Z* = −2.146, *p* = 0.03 and *Z* = −1.998, *p* = 0.05, respectively) (Figure 8A). The Micro-Focal strategy was the most used one here as in the above experiments.

The analysis of sensorimotor strategies according to geometric shape showed that all shapes were mainly recognized with the Micro-Focal strategy (square: 37.2%; circle: 36.7%; triangle: 32.2%). Mixed strategies were secondly employed (square: 23.3%; circle: 26.7%; triangle: 27.1%). Square and Circle were also frequently recognized with Micro-General strategy (23.3 and 20%, respectively) (Figure 8B). This suggests that Micro-Focal strategy allowed the participants to obtain relevant information to recognize each shape (for instance, for the square, the presence of straight sides and right angles; for the triangle, a tip on the top; and for the circle, a curved perimeter).

The analysis of the sensorimotor strategies according to trials order showed that in 1/3 and 2/3 of trials Micro-Focal strategies were used most frequently (38.2 and 36.9%, respectively). However, in the last third of trials, 3/3, Mixed strategies were the most employed (34.6%). This last strategy was most employed in the end of the test that in the beginning (*Z* = −2.588, *p* = 0.008), its use was increasing as the test progressed. The others strategies showed no significant changes along the trials (Figure 8C).

There are no previous studies that investigate in detail the movements that participants make while they echolocate. However, some studies highlight some features related to the results obtained here. Hausfeld et al. (1982), in the shape recognition study mentioned above, noted interesting differences between movements made by each participant. A blind person, who had echolocation skills, performed sweeping movements with his head while he emitted the echolocation signal. Also Milne et al. (2014), as mentioned, indicated that these movements were similar to eye movements that sighted persons make when scanning a scene.

#### Phenomenal data

Participants described their experience as auditory with reference to: (a) changes in the sound intensity, (b) changes in sound pitch (c) presence of a second sound added to the ST original sound. Also some participants compared their experiences on the test with everyday situations that involve strong attention in auditory or visual experiences (in Supplementary Material we present experience descriptions and related phenomenal data).

The different sounds properties mentioned here by participants could be related to psychoacoustic aspects involved in echolocation. The change of pitch of sound signals (Bassett and Eastmond, 1964; Bilsen and Ritsma, 1969/70; Arias and Ramos, 1997), the ability to detect spatial information contained in reflections (Saberi and Perrott, 1990; Arias, 2009), and also, perception of subtle changes in the intensity of sounds in the presence of an object (Schenkman and Nilsson, 2011; Milne et al., 2014) have been postulated as possible underlying auditory mechanism of human echolocation.

## Discussion

Participants equipped with three different SSDs, with minimum information about how each device worked and no previous training, were able to develop the necessary skills to recognize simple geometric shapes that they could not see directly. The participants' task was to indicate which shape was in front of him/her, from a limited set of previously known shapes, presented in perceptually simplified scenarios. We studied and classified the sensorimotor strategies employed by the participants. These shared some common features for all three devices but they also showed marked characteristics proper to each device.

### General performance

The response of the participants in all experiments showed functionally equivalent results regardless of SSD used: the hit scores obtained were similar and a learning effect was evident in each test. These results provide evidence that participants are able to progressively acquire sensorimotor skills to recognize simple geometric shapes with similar sensorimotor strategies regardless of the device concerned. This is consistent with previous results: it is possible, using only sound stimuli coupled to self-generated movements, to perform typical visual tasks (Proulx et al., 2008; Abboud et al., 2013; Viaud-Delmon and Warusfel, 2014). In fact, in a similar study, Bach-y-Rita et al. (1998), evaluated the performance of sighted adults in a geometric shape recognition task with a new version tactile-to-visual TVSS, a traditional visual-to-tactile SSD. In that study, participants had to distinguish three shapes (square, circle, and triangle), and their performance levels reached 79.8% of hits, a higher score than the one obtained here probably due to the greater experience that those participants had (a familiarization and a practice period up to a certain skill level). In our study, participants experienced only a brief familiarization with the task.

To varying degrees, the participants of this study demonstrated that they were able to perceive some physical properties that allowed them to recognize simple geometrical shapes. Even though results indicate a rapid development of skills to recognize shapes, it should be noted that the perceptual scenarios of experiments were fairly simplified. This choice was made to ensure enough control over measured variables while allowing the development of appropriate sensorimotor patterns with different SSD.

It would not be adequate to immediately extrapolate these results to natural settings. However, it is also the case that the task of reliably distinguishing between shapes does not reduce to the classification of local stimuli and that a form of integration is necessary and indeed always observed. The perceptual task was not defined only by the simplicity (relative to our normal vision) and geometrical differences between the shapes but also depends strongly on the SSD. In all cases sensory stimulation on its own is highly ambiguous (devices 1 and 3 provide on-off stimuli, device 2 provides highly contextual complex sound patterns) and must be integrated over time with active strategies and proprioceptive information. To put it in computational terms, all cases correspond to type 2 tasks as classified by Clark and Thornton (1997), i.e., tasks that cannot be solved by moment-to-moment sensory information and must rely on learned strategies for data gathering. As the results show, some geometrical shapes were easier to recognize than others depending on the device. In Experiments 1 and 3, the triangle was the most recognizable figure, whereas in Experiment 2 it was the square. These differences are likely due to how the devices work. Both the minimalist device (Invisible shapes discoverer) as the Sonic Torch involve local information gathering on the shape which is massively ambiguous in itself, only through active movement participants could get information about the shape of the figure. Determining local features of the shape become relevant only with respect to these strategies and once they are sufficiently developed. For instance, probably, the sensorimotor patterns developed in experiments 1 and 3 allowed to search for and easily distinguish local details like the tip of the triangle. By contrast, the vOICe gathers non-local information by the automatic sweeps it performs, which give at first no clear indication of the shape involved unless it is complemented by an active strategy. As mentioned above, the particular soundscape generated by the square was easily distinguished.

It is the need to actively construct such strategies that even for simple shapes (for normal vision) make all of these examples cases of real perception as opposed to the discrimination of unambiguous local and momentary stimuli.

### Sensorimotor strategies

Classical literature on perceptual strategies has described that there are many properties of the external world that can be obtained by both vision and haptic perception, through certain ways of sampling information which can be classified as parallel strategies (explore items all at once) and serial strategies (explore items one at a time) (Treisman and Gelade, 1980; Lederman and Klatzky, 1987; Lederman et al., 1988). The sensorimotor strategies described in these experiments can be categorized as variants of the serial strategies. This is probably due to the difficulty involved in these novel tasks, which has typically been associated with serial strategies where the person must explore the items one by one to find out whether they possess the target property. Minimalist SSDs, since they use binary information, allow only the development of serial strategies.

In general, the strategies identified in each experiment were similar, but they also show some particular, device-dependent features. The main differences across devices were related to the type of sweep. In the tests with the minimalist device (Experiment 1) and with echolocation (Experiment 3) the movement patterns made by participants were similar, that is: regardless of the effector used (head or hand, respectively) the trajectories were formed by sweeps, which could be large or small. The path drawn by these sweeps was zigzagging, allowing the participant to enter and leave the area of the figure. By contrast, in the test with the vOICe device (Experiment 2) movement patterns were composed mostly of jerky, small amplitude moves that began and ended with a period of relative stasis, like a fixation. The trajectories of these sweeps formed very diverse paths not necessarily in zigzag fashion. These differences are very likely due to how each device works. With the minimalist device, the user is continuously sensing information: every time she or he comes in contact with the object area a stimulus is heard. Similarly, in the echolocation test the participant, assisted with the Sonic Torch, emits an echolocation signal continuously and reflections are heard whenever the signal contacts the object area. In contrast, the vOICe device works by capturing and scanning optical images that are transformed in a sound pattern that lasts 1 s. If the user moves the head during the reproduction of a sound pattern he/she loses the spatial reference of what he/she is hearing. Experiment 2 shows that participants implicitly learned to engage with this way of sensing information. During fixations that occurred between the head movements they listened to the sound pattern of each optical scan. Participants moved their head when the vOICe finished the built-in image scan.

Participants in Experiments 1 and 3 had to generate a scan themselves in order to allow them to know when they were inside the shape or outside of it. This accounts for their perceptual trajectories describing zigzag movements that continuously cross the boundary of the shape. By contrast, participants with the vOICe device obtained information from a “perceptual window” (determined by the images that the camera captures) that could include the shape and its background. The user could not get information like “I'm inside” or “I'm outside” of the shape. With the vOICe, sweep trajectories moved the scanning “window” in relation to the object. For example, moving the window upward would eliminate information about the shape base, and so on.

Regarding to exploration mode, two well-defined styles were observed: generalized and focalized. In generalized exploration a participant conducts movements over the entire object surface or perimeter. By contrast, in focalized exploration, a participant makes his/her movements just over specific regions of objects to try to deduce its shape using local features (like the tip at the top in the case of a triangle). Both forms of exploration were observed in the strategies developed with the three types of SSD. These exploration modes are related with the exploration procedures described by Lederman and Klatzky (1987) in hand movements during haptic object perception. The exploratory procedures are considered as stereotyped movement determined by the object properties. The properties related to object structure are classified as global or exact shape information. In the exploratory procedure to obtain global shape information the effectors seek be in contact with as much of the envelope of the object as possible. This is useful to acquire general information about the object. Furthermore, in the exploratory procedures to obtain exact shape information the effectors movements are realized within a segment of the object contour, shifting direction when the contour ends. In this case, this is useful to perceive a particular salient dimension. In accordance with this classification, the generalized exploration mode described here corresponds with the exploratory procedures for obtaining global shape information, while the focalized exploration corresponds with the exploratory procedures to obtain exact shape information.

Based on these results, we suggest that there is a component of the sensorimotor patterns mainly related to the device, which in this experiment is manifested predominantly in the sweep type; and there is another component related properly with the recognition shape task, predominantly manifested in the exploration mode. In this sense, we consider that the sweeps features account for the presence of *apparatus-based* SMCs, i.e., forms of interactions allowed/constrained by the device to obtain useful information. While the exploration features indicate the presence of *object-related* SMCs, i.e., series of interaction patterns useful to recognize each shape.

Any SMC is a regularity involving always a relation between the agent and object. There are no canonical SMCs related to the sensory apparatus or related to the objects. For this reason, the approach of this study aims to highlight and compare basic characteristics of each SMC. In all cases a combination of both kinds of SMCs combine into a sensorimotor strategy and, in general, the contributions of apparatus and object-related SMCs will not always be easy to disentangle. In our case, the most frequently employed sensorimotor strategies, regardless of SSD, were Micro-Focal strategies, involving small movements on certain parts of the perimeter of the object. This would suggest that these types of interactions are preferred modes to obtain information about of the geometric shape of objects and correspond to the global functional aspects of the task given the set of shapes used and the aspects of the configuration common to all experiments.

In concordance with Maye and Engel (2012), we postulate that both kinds of contingencies are involved at all times in perceptual tasks, because one implies the other. It is possible to distinguish them only by performing an analysis in different time scales. The *apparatus-related SMCs* are responsible for the basic sense of information, while the *object-related SMCs* coordinated the former in complex sensorimotor sequences.

### First-person data

Based on the phenomenological reports of two late blind expert users of the vOICe device, Ward and Meijer (2010) suggest that an extensive use of this kind of auditory-to-visual SSD may provoke visual experiences. Also, many studies indicate that prolonged use of a SSD may generate synesthetic experiences (Proulx and Stoerig, 2006; Proulx, 2010; Farina, 2013; Renier and De Volder, 2013; Ward and Wright, 2014; Auvray and Farina, in Press). Proulx (2010) suggests that to achieve this kind of perception it would be necessary to have a given sensorimotor expertise with the device.

Nevertheless, participants in this study associated their perception mostly to auditory experiences. This may possibly be due to the brief training they had with the devices. Other experiments involving only short-term exposure to SSDs lead to similar conclusions (Auvray et al., 2007a; Thaler et al., 2014). It is possible to conjecture that in the early development of skills using SSDs the experiences of the user correspond largely to the mastery of apparatus-related SMCs. These SMCs are determined by the sensor and effector features (O'Regan and Noë, 2001). It would make sense to expect that as the user gains sensorimotor expertise his/her experience comes closest to the information generated from object-related SMCs and he/she pays less attention to apparatus-related SMCs. This progression from one kind of SMCs to another could account for some of the cases where first-person reports indicate perceptual experiences corresponding to the functional modality of the task and not to the kind of stimulus delivered by the device.

The evidence of progressive learning undergone by the participants during the task and with each SSD is an indicator of the achievement of a mastery of the novel SMCs. Such mastery, according to the theory, is constitutive of early forms of perception. We infer based on phenomenological data that the skill of participants was mainly determined by their familiarization with the active use of each SSD, i.e., their mastery was more closely related to apparatus-based SMCs. The mastery of object-related SMCs would in addition indicate capabilities involved in more complex perceptual tasks, such as recognizing objects in a wide set of possibilities, or, different versions of a same class of object. This more in-depth mastery could be tested in other experimental situations with subjects undergoing more extensive training with the SSD and solving diverse tasks, such as “using” or manipulating a variety of geometrical shapes presented simultaneously.

Consequently, metacognitive data reflecting the conscious monitoring or control of sensorimotor strategies, indicate that participants made use of inferences and deductions at least at some stages. This type of cognitive strategies are useful for the achievement of mastery of SMCs, mainly the apparatus-related ones, and it is characteristic of many sensorimotor learning processes, especially those that involve SSDs. Whenever a novel sensorimotor task is learned the participant typically thinks about how it is (or should be) carried out. The use of such cognitive strategies is replaced by a more embodied and unreflective usage of the device as some degree of expertise is achieved. This effect was observed in Auvray et al. (2007a), where participants' perception, as their learning progressed, passed from a form of deductive reasoning to a more intuitive and immediate apprehension of what was perceived.

This correspondence between type of SMCs and perceptual experience would be consistent with the learning process proposed by Bach-y-Rita (1972) and Auvray (2004). Initially, users are conscious of the interface device itself. Progressively, they begin to ignore the stimuli provided by the device and concomitantly they perceive an external object located out there in a distal space. The device becomes transparent when the sensorimotor contingencies have been assimilated and become second nature (Stewart and Khatchatourov, 2007).

For these reasons, we believe that while the development of more generalizable forms of object-related SMCs underlie the constitution of more direct forms of object perception and that such development requires more diverse tasks and training, the use of metacognitive strategies is not in itself evidence that no mastery at all is achieved and that the task is resolved by some kind of cognitive discrimination. In fact the learning progression indicates the achievement of mastery of SMCs as required by the experimental set-up and sufficient for resolving the task and therefore of the emergence of perceptual skills mediated by each SSD, even if circumscribed by the conditions of the experiment.

### The nature of perception in sensory substitution

The analysis of SMCs in our experiments suggests that apparatus-related SMCs are associated with the acquisition of novel skills allowing a user to engage with the new interface. Conversely, in our study, *object-related SMCs* seem to remain associated with earlier abilities, i.e., prototypical ways of interaction between agent and environment.

Recently Di Paolo et al. (2014) have postulated that, in the framework of sensorimotor approach, perceptual learning originates always in pre-existing sensorimotor structures, which undergo a process of equilibration under novel conditions. New patterns of interaction are generated on the basis of adapting existing SMCs to a new context. In this case, the form of the novel sensorimotor loop would seem to be largely driven by the properties of the SSD, while the fact that a particular kind of closure is sought for during adaptation, corresponds to the functional aspects of shape recognition (e.g., exploring contours, determining shape size, exploring local features).

In general, the perceptual trajectories that participants performed with the SSDs were similar to the known trajectories of visual exploration, mainly composed of a series of straight movements qualitatively similar to eye-saccades. Putting aside the physiological differences between the effectors involved (for example, ocular saccades are much faster than the movements of the head or hand) in either case this pattern of movements would allow quickly guiding the attention only to the regions of interest. Also, other authors have reported the same similarities between the movements made by participants equipped with a SSD or when they echolocated with visual saccades (Chekhchoukh et al., 2013; Milne et al., 2014; Ward and Wright, 2014). It makes sense to assert, following O'Regan and Noë (2001), that the nature of perception obtained with a SSD is determined by a recreation of existing SMCs patterns analogous to patterns proper to the modality replaced by the device. How a visual-to-auditory SSD user interacts with his environment is based on forms of visual interaction in similar environments. But as we have seen, the phenomenal aspects of perceptual experience may still be dominated by apparatus-related SMCs depending on the level of skill incorporated by the user. Some of these forms of sensorimotor interactions may correspond to even more basic strategies than those involved in the visual perception. The structure of sensorimotor visual patterns has been linked to haptic perception patterns. As noted by Merleau-Ponty (1968) and MacKay (1962, 1973) (both cited in O'Regan and Noë, 2001) vision could be considered as a kind of *palpation* with the eyes or like a giant hand that samples the environment. Gapenne (2010) has also suggested that saccadic eye movements, as well as movements performed with visual-to-auditory SSD, are rooted in movements done by hand in haptic exploration. Briefly, Gapenne's thesis assumes that while haptic perception involve the stimulation of tactile receptors associated with movements of the body which also stimulate the proprioceptive system, the contact between fingers and the object is felt as a resistance offered by the object to these movements. In the case of vision and also in sensory substitution in the absence of such direct contact the constitution of this experience is based on implicit kinesthetic knowledge. In the perceptual constitution of distal objects a quasi-resistance of a distal object is constituted through the exteroceptive guidance of exploratory activity. In vision, for example, oculomotor exploration is allowed or constrained by the morphological singularities of the object. In this sense, fixations and saccades that an individual makes over an object to recognize it are equivalent to the resistance received in haptic exploration.

The equilibration of these basic sensorimotor interactions to novel conditions involves changes in the SMCs structure. The constraints provided by each SSD lead to novel components in those contingencies. As suggested by Auvray and Myin (2009), Kiverstein and Farina (2012), Farina (2013) and Deroy and Auvray (2014), perception with a SSD is, therefore, a new way of perceiving.

We note that this general remark has a special significance for the case of echolocation (Experiment 3). Human (non-enhanced) echolocation, as used by many blind people, shows some of the same characteristics found in the case of the Sonic Torch. Echolocation is a closed loop behavior where the agent modulates action to control perception (Stoffregen and Pittenger, 1995). Without the self-generation of sounds and head movements there is no possibility of perception. In this way, echolocation can be seen as a natural sensory substitution strategy (Arias et al., 2011) and may be studied, together with other cases of sensory substitution, with the same concepts and tools.

## Conclusion

Our study provides empirical support for SMC theory. As far as we know, this is the first study in sensory substitution that analyzes and compares the emergence of SMCs with different types of SSDs. The results provide evidence that sheds light on: sensorimotor integration in an active perceptual task, the role of the voluntary execution of movements in the sensorimotor learning, and the relationship between existing sensorimotor structures and the achievement of a new sensorimotor expertise. The latter observation supports new theoretical proposals about learning of SMCs as developing out of pre-existing equilibrated sensorimotor strategies through processes of adaptation (Di Paolo et al., 2014).

Likewise, the results support critical perspectives on classical conceptions of perceptual modalities. These perspectives emphasize the relevance of ecological, embodied and functional aspects of perception – e.g., the localization and recognition of objects – rather than the input channels or sensory organs concerned, as the main determinants of perceptual modalities (see for example, Gibson, 1966; Pascual-Leone and Hamilton, 2001; McGann, 2010).

The comparative use of SSDs can help us understand how perceptual spaces are structured through the emergence of different kinds of SMCs, those related to the apparatus and those related to object (or functional task). In comparison with related work, our results allow us to speculate that perceptual experience may be dominated by apparatus-related SMCs at first and progressively by object-related SMCs, as the user increasingly incorporates the SSD. This can account for the divergence of reports across different studies concerning the first-personal description of the perceptual modality involved. Interestingly, task performance is already high at the early stages of this progression, as if the postulated progression was a form of refinement of a sensorimotor task that already works functionally. Our aim in future work is to further study how sensorimotor strategies emerge, stabilize and become refined as the contingencies of the SSD get further mastered and incorporated into the perceptual skill set of the agent.

### Conflict of interest statement

The authors declare that the research was conducted in the absence of any commercial or financial relationships that could be construed as a potential conflict of interest.
