# Supplementary material for: Sensorimotor strategies for recognizing geometrical shapes: a comparative study with different sensory substitution devices
Source: Front Psychol. 2015 Jun 9;6:679. doi: 10.3389/fpsyg.2015.00679 (PMC4460306; doi:10.3389/fpsyg.2015.00679)
Supplement: Supplementary file 1 [file DataSheet1.PDF]

Supplementary Material

Sensorimotor strategies for recognizing geometrical shapes: a comparative study with different sensory substitution devices

Fernando Bermejo[1,2], Ezequiel Di Paolo[3,4,5], Mercedes X. Hüg[1,2,6] and Claudia Arias[1,2,6]

[1]Centro de Investigación y Transferencia en Acústica (CINTRA), Universidad Tecnológica Nacional, Facultad Regional Córdoba - Unidad Asociada de CONICET, Argentina.
[2]Facultad de Psicología, Universidad Nacional de Córdoba, Argentina
[3]Ikerbasque, Basque Foundation for Science, Spain
[4]IAS-Research Center for Life, Mind, and Society, Department of Logic and Philosophy of Science, University of the Basque Country, San Sebastián, Spain
[5]Centre for Computational Neuroscience and Robotics, Department of Informatics, University of Sussex, Brighton, UK
[6]Consejo Nacional de Investigaciones Científicas y Técnicas (CONICET), Argentina

*** Correspondence:** Corresponding Author, Centre for Research and Transfer in Acoustics, Unit Associated of CONICET, National Technological University, Córdoba Regional Faculty, Maestro M. Lopez esq. Cruz Roja, Córdoba 5000, Argentina.
fbermejo@psyche.unc.edu.ar

1. Examples of perceptual trajectories according to different Sensorimotor Strategies

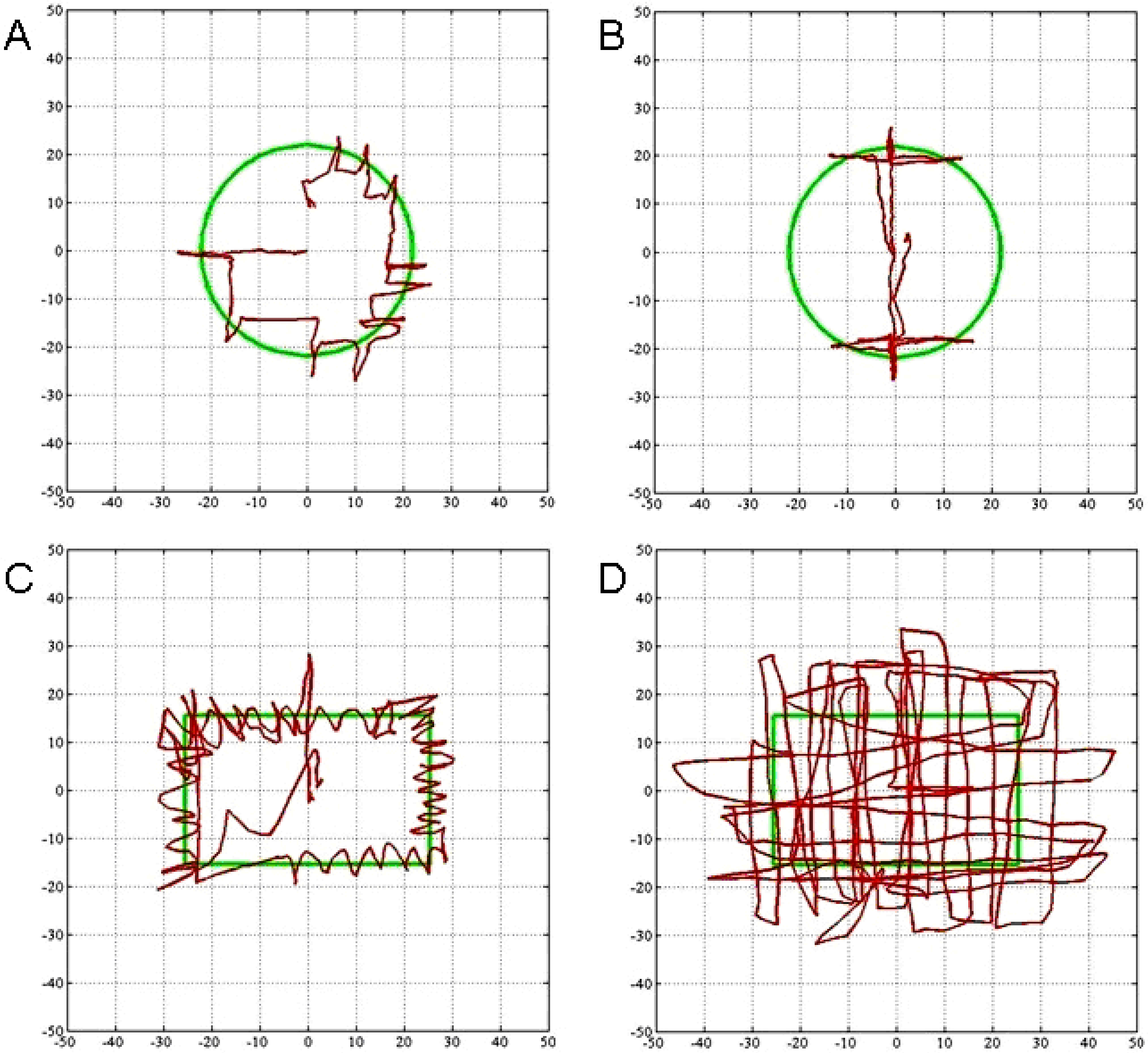

Figure 1. Front view of perceptual trajectories performed by participants in Experiment 1. The horizontal and vertical axes represent the azimuth and elevation (in deg), respectively. The red line corresponds to perceptual trajectory; green lines represent the shape to recognize. The perceptual trajectory of graph A corresponds to the Sensorimotor Strategy Micro-Focal, perceptual trajectory of graph B to Macro-Focal, perceptual trajectory of graph C to Micro-General and perceptual trajectory of graph D to Macro-General.

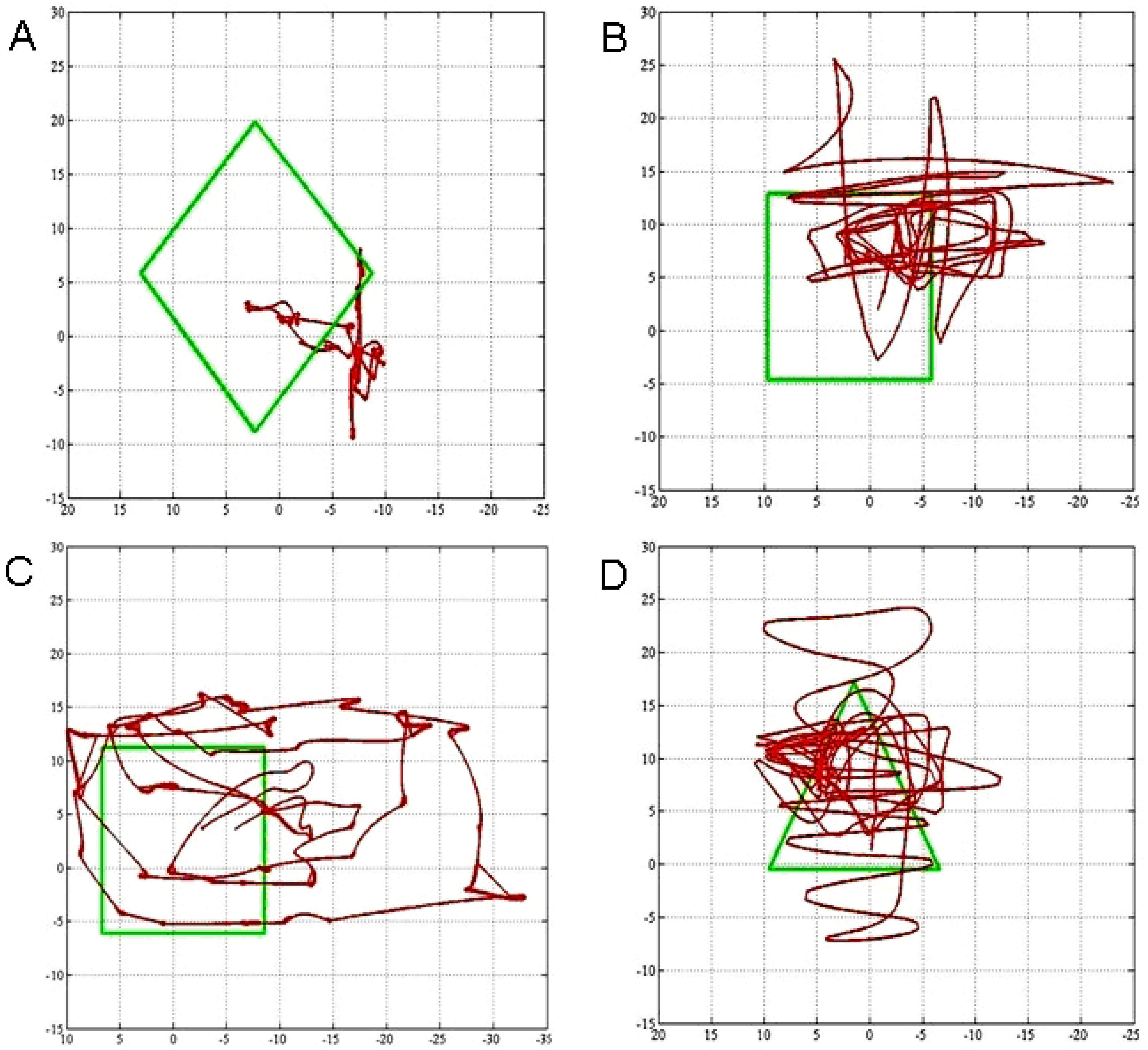

Figure 2. Front view (as in Figure 1) of sensorimotor trajectories performed by participants in Experiment 2. The perceptual trajectory of graph A corresponds to the Sensorimotor Strategy Micro-Focal, perceptual trajectory of graph B to Macro-Focal, perceptual trajectory of graph C to Micro-General and perceptual trajectory of graph D to Macro-General.

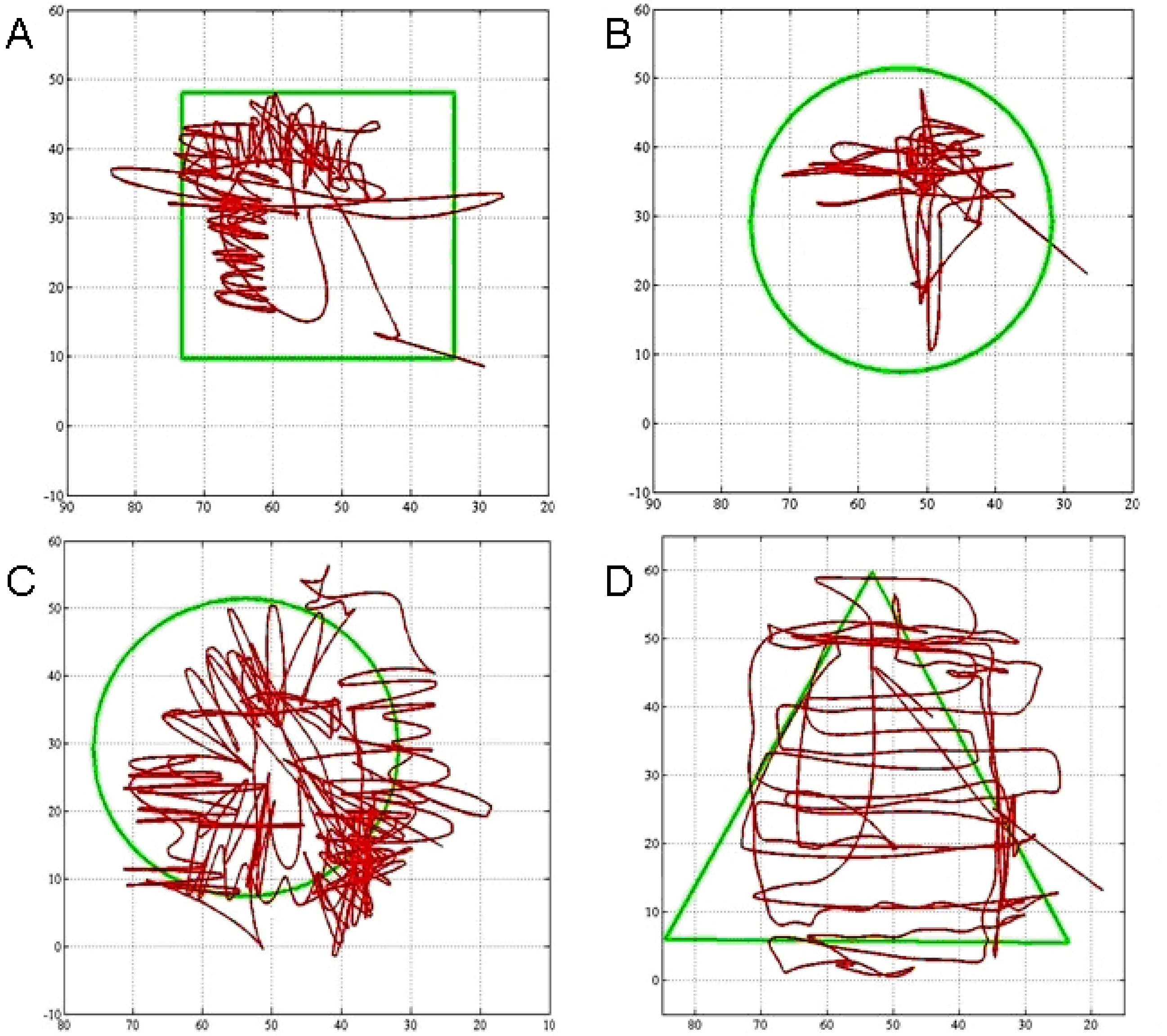

Figure 3. Front view (as in Figure 1) of sensorimotor trajectories performed by participants in Experiment 3. The perceptual trajectory of graph A corresponds to the Sensorimotor Strategy Micro-Focal, perceptual trajectory of graph B to Macro-Focal, perceptual trajectory of graph C to Micro-General and perceptual trajectory of graph D to Macro-General.

2. Experiences description and related phenomenal data

Table 1. Phenomenal data from Experiment 1

Visual imagery

Participants imagined they saw the object as long as they develop an action to discover it.

Phenomenal data

I thought there was a blank page, noise wrote it. When it kept ringing, stopped writing (Participant Nº 2).
I imagined I would paint [the shapes] with the primary colors for example the circle was red (Participant Nº 6).
It was more visual, as if I projected something like a laser (Participant Nº 3).

Table 2. Phenomenal data from Experiment 2

**Global auditory perception**<br><br>Recognition of particular sound signatures of each shape<br><br>**Phenomenal data**<br><br>*When I heard "psh psh" it was a triangle. I could distinguish the triangle from the rhombus because I heard a sound rather like "piun piun" with the triangle and not so much like "pin pin". The rhombus was also more like a softer flashing sound. The square was like a dry line* (Participant Nº 9).<br>*Once you've learned the sound of each shape, you didn't need to move and you could listen to each line. You could listen to it all at once and confirm it with movements. There were 3 clearly distinct sounds* (Participant Nº 8).
**Fractionated auditory perception**<br><br>Progressive construction of the geometric shape based on the search particular clues sound<br><br>**Phenomenal data**<br><br>*I explored the sounds, which were very diverse … I think I was moving too much; I couldn't recognize [the shape] if I wasn't moving. I didn't have a mental image of the shape, so I had to build it progressively* (Participant Nº 11).<br>*I used auditory perception without first making images. I tried to listen carefully. At first I didn't imagine the shapes, but then yes, in order to give an answer* (Participant Nº 10).

Table 3. Phenomenal data from Experiment 3

**Auditory Sensations**
**Sound level**<br>To detect changes in sound intensity (property for classifying sounds from weak to strong)<br>**Phenomenal data**<br>*When I passed over the object [the sound] was louder; the volume went down where there was nothing* (Participant Nº 14)
**Sound pitch**<br>To detect changes in the pitch of the sound (property for classifying sounds from high-pitched to low-pitched).<br>**Phenomenal data**<br>*I thought there was a higher frequency when I entered the shape* (Participant N° 7)
**Adding sounds**<br>To detect the presence of a second sound added to the ST sound, i. e., sound reflections.<br>**Phenomenal data**<br>*When the shape wasn't there the sound would go away, when it was there, it would come back* (Participant Nº 11)
**Everyday situations references**<br>Comparisons with everyday experiences related to auditory skills or visual type sensations associated with movements performed<br>**Phenomenal data**<br>*It was like tuning the guitar, you could hear something else, something left over, a vibrator …* [in the case of the triangle] *at the base of the shape this left over should take longer* (Participant Nº 2).<br>*It was like painting, like when you're making a drawing. The movement was like a drawing, but in fact I was exploring, as if discovering something* (Participant Nº 16).

3. Protocol for training practice with the vOICe

Participants performed a training practice of about 30 minutes to develop minimal abilities in handling the vOICe device. The training consist on a set of simple tasks that the participants must resolve in a sequential order: 1) to detect the presence of a 5 cm square positioned in front of them (Figure 4); 2) to locate the same square in different positions (right, center and left); 3) to recognize the orientation of a 10 cm white bar (horizontally, vertically, inclined bottom-up or inclined top-down) (Figure 5); 4) to identify the orientation of 2 bars of 15 cm that form a right angle (with the base up, the base down, the vertex to the left or the vertex to the right) (Figure 6). In each case, participants were sat down in the chamber, blindfolded and equipped with the vOICe. When they performed correctly the same task 3 times, they moved on to the next step.

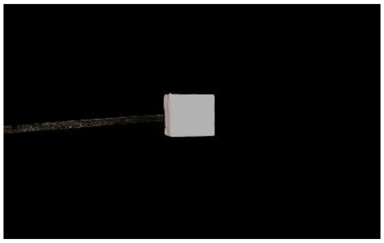

Figure 4. Object fixed in a black rod.

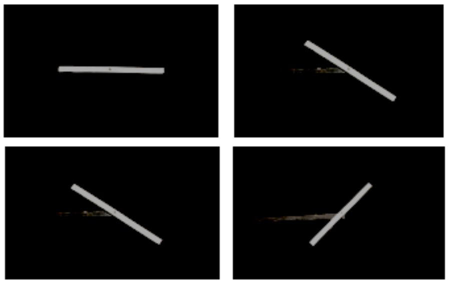

Figure 5. Possible bar orientations.

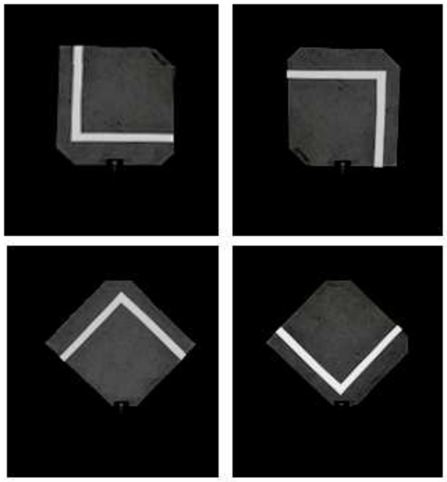

Figure 6. Possible two bars orientations.
